# Australian Gay and Bisexual Men’s Attitudes to HIV Treatment as Prevention in Repeated, National Surveys, 2011-2013

**DOI:** 10.1371/journal.pone.0112349

**Published:** 2014-11-11

**Authors:** Martin Holt, Toby Lea, Dean A. Murphy, Jeanne Ellard, Marsha Rosengarten, Susan C. Kippax, John B. F. De Wit

**Affiliations:** 1 Centre for Social Research in Health, UNSW Australia, Sydney, New South Wales, Australia; 2 Australian Federation of AIDS Organisations, Sydney, New South Wales, Australia; 3 Australian Research Centre in Sex, Health and Society, La Trobe University, Melbourne, Victoria, Australia; 4 Department of Sociology, Goldsmiths, University of London, London, United Kingdom; 5 Social Policy Research Centre, UNSW Australia, Sydney, New South Wales, Australia; 6 Social and Organizational Psychology, Utrecht University, Utrecht, the Netherlands; Rollins School of Public Health, Emory University, United States of America

## Abstract

**Objective:**

Assess the acceptability of HIV treatment as prevention and early antiretroviral treatment among gay and bisexual men in Australia and any changes in attitudes over time.

**Methods:**

National, online, cross-sectional surveys of gay and bisexual men were repeated in 2011 and 2013. Changes in attitudes to HIV treatment over time were assessed with multivariate analysis of variance. The characteristics of men who agreed that HIV treatment prevented transmission and thought that early treatment was necessary were identified with multivariate logistic regression.

**Results:**

In total, 2599 HIV-negative, untested and HIV-positive men participated (n = 1283 in 2011 and n = 1316 in 2013). Attitudes changed little between 2011 and 2013; most participants remained sceptical about the preventative benefits of HIV treatment. In 2013, only 2.6% of men agreed that HIV treatment prevented transmission; agreement was associated with being HIV-positive, having an HIV-positive regular partner, and having received HIV post-exposure prophylaxis. In contrast, 71.8% agreed that early antiretroviral treatment is necessary; younger men were more likely and HIV-positive men and participants with HIV-positive partners were much less likely to agree with this.

**Conclusions:**

Promoting the individual health benefits of HIV treatment rather than its preventative benefits remains more acceptable to Australian gay and bisexual men.

## Introduction

The idea of using antiretroviral treatment (ART) as a form of HIV prevention (treatment as prevention or TasP) has gained considerable prominence since the results of the HPTN 052 randomised controlled trial showed that ‘early’ treatment (initiating ART above 350 CD4 cells/mm^3^) and sustaining an undetectable viral load dramatically reduced HIV transmission between serodiscordant heterosexual partners [1], [2]. More recently, preliminary findings from the PARTNER study suggest that TasP may be equally efficacious in preventing transmission between men who have sex with men (MSM) [3].

In 2013 the World Health Organization issued consolidated guidelines on treating and preventing HIV which recommended that ART should be initiated when a person’s CD4 count drops below 500 cells/mm^3^ [4]. The guidelines also recommended that all HIV-positive people in serodiscordant (different HIV status) relationships should be offered ART, regardless of clinical stage, in order to prevent transmission to the HIV-negative partner. The recommendation that ART should be initiated early has recently gained additional support in a subsequent analysis of the HPTN 052 data, which showed that patients who received early treatment experienced significantly fewer HIV-related clinical events [5].

While international treatment guidelines have gradually shifted to embrace the early initiation of ART, it is far from clear whether this approach will be effective in curbing HIV transmission in countries that already report relatively high levels of HIV testing and treatment uptake [6]. There has also been a notable lack of research on the acceptability of the TasP approach to affected communities [7], [8]. This raises questions about how easily and effectively TasP can be implemented if levels of community support and interest are not well understood [9].

In 2011, we conducted a national survey of Australia’s primary affected population, gay and bisexual men, looking at attitudes to antiretroviral-based prevention [10], [11]. The survey was conducted six months before the first HPTN 052 results were published [1]. We found generally positive attitudes towards the health benefits of HIV treatments but scepticism about the benefits of ART in preventing HIV transmission, particularly among HIV-negative men. These findings suggested that implementing TasP in Australia would require engagement with gay and bisexual men to address their concerns.

In 2012, the HIV strategy for Australia’s most populous state, New South Wales, embraced the idea of TasP and encouraged a greater focus on HIV testing to reduce undiagnosed infection and the use of HIV treatment to reduce transmission [12]. Queensland adopted a similar strategy in 2013 [13], and the recently released Seventh National HIV Strategy builds on these policies and proposes that 90% of people living with HIV should be on treatment by 2017 [14]. These strategies have led to the expansion of HIV testing through, for example, the development of community-based rapid HIV testing services. Public campaigns aimed at gay men (notably the ‘Ending HIV’ campaign that originated in New South Wales) are heavily promoting routine HIV testing and the benefits of early treatment uptake. Regulatory policies have also been revised to allow, for example, doctors to prescribe publicly-subsidised ART regardless of CD4 cell count. The strategies continue to emphasise that the uptake of HIV testing and treatment should be voluntary and informed. Within this shifting policy context, in 2013 we repeated a national survey to assess levels of support for TasP and the early initiation of ART and the characteristics of men who were supportive (or not) of these strategies.

## Methods

### Participants and procedures

Data were collected as part of the PrEPARE Project, a study of Australian gay and bisexual men’s attitudes to antiretroviral-based HIV prevention technologies [10], [11]. The study design was approved by the University of New South Wales Human Research Ethics Committee (ref. HREC 11034). National online surveys of gay and bisexual men were conducted in April-May 2011 and June–July 2013. We used the NETQ online survey platform (NetQuestionnaires Nederland BV). The survey was promoted with paid advertising on Facebook and notices sent to email lists aimed at gay and bisexual men. Participants from the 2011 survey who had agreed to be contacted by email were invited to participate in the 2013 survey. Potential participants were directed to the survey website, http://prepareproject.csrh.org, which explained the purpose of the study and provided access to the online questionnaire. Participants were eligible if they were at least 18 years old, male, lived in Australia and were gay, bisexual or other MSM. Eligibility was confirmed with the first questions in the survey; if the participant indicated they were ineligible, the survey stopped and the participants were thanked for their time. Completing the online survey was taken as evidence of consent. No incentive was offered for participation.

### Measures

Wherever possible, the same questions were used in both survey rounds. Both surveys included common questions about demographics, sexual practices with men, relationships, HIV testing and HIV status, adapted from behavioural surveillance questionnaires [15].

The questionnaires contained a range of Likert-type attitudinal items (each scored from 1 = strongly disagree to 5 = strongly agree). Five attitudinal variables related to HIV treatments and TasP were common to both rounds. These items were derived from research on HIV treatment optimism [16]. In the 2013 survey two new reliable scales were identified. The first scale, HIV treatment prevents transmission (Cronbach’s α = 0.65), contained three items: an HIV-positive person who is on HIV treatments is unlikely to transmit HIV; a person with an undetectable viral load cannot pass on HIV; if every HIV-positive person was on treatment the HIV epidemic would be over. The second scale, early HIV treatment is necessary (α = 0.72), contained three items: people should start HIV treatment as soon as they are diagnosed; people should delay treatment until it is absolutely necessary (reverse scored); HIV-positive people should go on treatment to protect their partners. We note that the last item in the early HIV treatment is necessary scale implies protection from transmission (which suggests overlap with the idea of TasP). However, it may also imply a broader sense of protection i.e. protecting both partners from the adverse effects of the HIV-positive partner becoming ill from HIV. For this reason, we believe that the two scales are sufficiently distinct. Scale scores were a mean of the items within the scale (from 1 to 5), with a score of ≥4 indicating positive agreement e.g. participants who scored ≥4 on the HIV treatment prevents transmission scale were classified as agreeing that HIV treatment prevents transmission. The cut-off of 4 was used as this was the point on the ordinal scale that indicated agreement with the subject of the scale i.e. 4 = agree on the Likert scale. This was the scoring method used in the other publications from the study [10], [11].

### Data analysis

SPSS Version 20 was used for data analysis. Statistical significance was set at *p*<0.05, unless otherwise specified. The characteristics of the 2011 and 2013 samples were compared using chi-square tests and t-tests to identify potentially confounding factors. We examined a standard set of demographic variables (e.g. age, education, employment status), variables that we have previously found to be associated with HIV risk and interest in antiretroviral-based prevention [11], and factors that reviews suggested might be associated with interest in TasP and early HIV treatment [7], [8]. Multivariate analysis of variance (MANOVA) was used to see if attitudes to HIV treatments had changed between 2011 and 2013, stratified by HIV status and controlling for potentially confounding factors. The characteristics of men in 2013 who agreed that HIV treatment prevents transmission and those who agreed that early HIV treatment is necessary were analysed with multivariate logistic regression to identify independent relationships with each scale. Unadjusted and adjusted odds ratios are reported.

## Results

In 2011, the online survey was completed by 1283 men, of whom 919 were HIV-negative, 122 were HIV-positive and 242 were untested or of unknown HIV status. In 2013, the online survey was completed by 1316 men, of whom 966 were HIV-negative, 93 were HIV-positive and 257 were untested or of unknown HIV status. Sample characteristics are shown in Table 1.

**Table 1:** Sample characteristics.

		2011 sample(n = 1283), %	2013 sample(n = 1316), %	t or χ^2^ (*p*)
Age	Mean in years (SD)	31.5 (10.9)	32.0 (11.1)	1.07 (0.29)
Sexual identity	Gay	92.3	91.6	5.90 (0.05)
	Bisexual	6.9	6.5	
	Other	0.9	2.0	
HIV status	HIV-negative	71.6	73.4	5.12 (0.08)
	Untested/unknown status	18.9	19.5	
	HIV-positive	9.5	7.1	
Country of birth	Australia	81.8	77.1	8.83 (0.003)
	Overseas	18.2	22.9	
State or territory	Australian Capital Territory	2.8	3.6	15.30 (0.03)
	Queensland	17.4	20.1	
	New South Wales	37.1	31.8	
	Northern Territory	0.4	0.4	
	South Australia	6.2	5.6	
	Tasmania	2.2	1.2	
	Victoria	26.1	29.1	
	Western Australia	7.9	8.2	
Residential location	Capital city	76.2	75.8	0.08 (0.96)
	Other city	10.0	10.1	
	Regional or rural area	13.8	14.1	
Employment	Full-time	59.5	55.9	13.32 (0.004)
	Part-time	10.8	10.2	
	Student	18.5	24.2	
	Unemployed/retired/other	11.3	9.7	
Education	Up to Year 12 (high school)	34.5	28.4	32.89 (<0.001)
	Trade certificate	21.7	16.6	
	Undergraduate degree	26.7	32.6	
	Postgraduate degree	17.1	22.3	
No. of male sex partners in past six months	None	9.5	10.3	6.90 (0.08)
	One	21.1	24.5	
	2–10	47.9	46.8	
	>10	21.4	18.4	
HIV status of regular male partner	No regular partner	47.5	44.2	10.28 (0.02)
	HIV-negative	40.3	43.8	
	Untested/unknown status	5.8	7.4	
	HIV-positive	6.5	4.6	
Anal intercourse with regular male partners(past six months)	No partner/no intercourse	35.9	35.1	0.43 (0.81)
	Consistent condom use	18.2	17.8	
	Any anal intercourse withoutcondoms	45.8	47.1	
Anal intercourse with casual male partners(past six months)	No partner/no anal intercourse	40.7	42.4	1.14 (0.56)
	Consistent condom use	31.1	31.1	
	Any anal intercourse withoutcondoms	28.2	26.5	
Ever received HIV post-exposureprophylaxis (PEP)	No	85.8	86.2	0.07 (0.79)
	Yes	14.2	13.8	

SD = standard deviation, t = t-test result, χ^2^ = chi-squared test result, *p* = probability.

The majority (82.8%) of the total sample from both survey rounds was recruited via Facebook advertisements and the study’s Facebook page. The remainder of respondents were recruited via advertisements on gay websites (7.5%), email lists (5.9%), word-of-mouth (2.5%) and other sources (1.3%). There was a small but significant difference in the proportion of respondents recruited via Facebook between 2011 and 2013 (79.7% vs. 85.8%; χ^2^ = 16.73, *p*<0.001).

The majority of the sample identified as gay, was born in Australia and lived in the capital city of their state or territory. Most were in full-time employment and over half had a university education. About a fifth reported more than ten male sex partners in the six months prior to survey, about half reported any anal intercourse without condoms with a regular male partner, and a quarter reported any anal intercourse without condoms with casual male partners. One in seven said they had received HIV post-exposure prophylaxis. Seventy-three percent of HIV-negative men reported having had an HIV test in the previous 12 months, while 91.4% of untested/unknown status men had never had an HIV test. Among HIV-positive respondents, 89.2% reported being on antiretroviral treatment in 2011 and 78.5% in 2013 (χ^2^ = 3.38, *p* = 0.06). The proportion of HIV-positive men with an undetectable viral load was 78.4% in 2011 and 74.2% in 2013 (χ^2^ = 0.52, *p* = 0.77).

There were some differences between the 2011 and 2013 samples (see Table 1). In the 2013 round more men said they were born overseas, a smaller proportion was recruited from New South Wales, a higher proportion of students took part, a higher proportion reported a university education and men were more likely to have a HIV-negative regular partner. All of these differences between the 2011 and 2013 samples were restricted to HIV-negative men (analysis not reported here); the profiles of untested/unknown status and HIV-positive men were similar in 2011 and 2013.

### Attitudes to HIV treatments in 2011 and 2013

Table 2 shows the participants’ mean scores on five attitudinal items concerned with HIV treatments included in both the 2011 and 2013 surveys. In general, HIV-negative and untested/unknown status men were sceptical about items related to TasP, disagreeing that an HIV-positive person who is on HIV treatments is unlikely to transmit HIV or that a person with an undetectable viral load is unlikely to pass on HIV. They also disagreed with the idea that they were less worried about HIV because of treatments. They were, however, neutral about whether it was difficult or easy to take HIV treatments.

**Table 2:** Attitudes to HIV treatments in 2011 and 2013, by HIV status.

		2011, M (SD)	2013, M (SD)	*F*	*p*	η_p_^2^
HIV-negative men	Because of HIV treatments I’m less worried about HIV infection than I used to be	2.27 (1.15)	2.26 (1.18)	0.48	0.49	<0.001
	An HIV-positive person who is on HIV treatments is unlikely to transmit HIV	1.92 (0.96)	2.18 (1.08)	8.68	0.003*	0.005
	A person with an undetectable viral load cannot pass on HIV	1.96 (0.94)	2.09 (1.05)	6.53	0.011	0.003
	It is difficult to take HIV treatments every day	3.02 (0.99)	2.97 (1.07)	1.86	0.17	0.001
	Taking HIV treatments is simple and straightforward	2.70 (0.96)	2.80 (1.08)	4.39	0.04	0.002
Untested/unknownstatus men	Because of HIV treatments I’m less worried about HIV infection than I used to be	2.33 (1.10)	2.31 (1.12)	0.04	0.85	<0.001
	An HIV-positive person who is on HIV treatments is unlikely to transmit HIV	1.90 (0.87)	1.98 (0.97)	0.84	0.36	0.002
	A person with an undetectable viral load cannot pass on HIV	2.19 (0.94)	2.05 (0.94)	2.59	0.11	0.005
	It is difficult to take HIV treatments every day	2.82 (0.87)	3.09 (0.99)	9.91	0.002*	0.02
	Taking HIV treatments is simple and straightforward	2.84 (0.85)	2.81 (1.01)	0.16	0.69	<0.001
HIV-positive men	Because of HIV treatments I’m less worried about HIV infection than I used to be	3.38 (1.24)	3.16 (1.35)	1.48	0.22	0.007
	An HIV-positive person who is on HIV treatments is unlikely to transmit HIV	2.73 (1.19)	2.68 (1.29)	0.09	0.76	<0.001
	A person with an undetectable viral load cannot pass on HIV	2.18 (1.04)	2.15 (0.98)	0.05	0.83	<0.001
	It is difficult to take HIV treatments every day	2.49 (1.12)	2.16 (1.12)	4.60	0.03	0.02
	Taking HIV treatments is simple and straightforward	3.60 (1.04)	3.73 (1.11)	0.81	0.37	0.004

Items were scored from 1 = strongly disagree to 5 = strongly agree. *F* = analysis of variance test result, *p* = probability, η_p_^2^ = partial eta-squared (effect size). *Statistically significant difference between 2011 and 2013 mean scores (*p*<0.01).

A factorial MANOVA was conducted to identify whether there were any changes in HIV-negative men’s attitudes to HIV treatments between 2011 and 2013 (see Table 2). The analysis controlled for country of birth, employment status, education level, and number of male sexual partners in the previous six months. The multivariate test showed a significant main effect for survey year (Wilks’ λ = .99, *F*[5, 1859] = 2.83, *p* = .01, η_p_^2^ = .008). Post hoc estimation indicated that the analysis was well powered to detect an effect (at 0.98) [17]. Bivariate main effects were examined because the overall test was significant, with a Bonferroni correction to the statistical significance level due to multiple comparisons (*p* = 0.05/5 = 0.01). At the *p*<0.01 level, only one statistically significant change was identified; HIV-negative respondents in 2013 were slightly less sceptical about the idea that an HIV-positive person on HIV treatments is unlikely to transmit HIV.

A one-way MANOVA was conducted on untested/unknown status men’s attitudes to HIV treatments (see Table 2), finding a significant main effect for survey year (Wilks’ λ = 0.97, *F*[5, 493] = 3.09, *p* = 0.009, η_p_^2^ = 0.03). Control variables were not included because there were no significant sociodemographic or behavioural differences between untested/unknown status respondents in 2011 and 2013. The analysis was well powered (at 0.87). One statistically significant change was identified; in 2013, untested/unknown status men were slightly more likely to agree that it is difficult to take HIV treatments every day.

As can be seen from the mean scores in Table 2, HIV-positive men slightly agreed with the idea that HIV treatments reduced their concern about HIV, and moderately agreed that taking HIV treatments was simple. They disagreed that it was difficult to take HIV treatments every day. There were sceptical about (they disagreed with) the TasP items: that an HIV-person on treatments is unlikely to transmit HIV and that a person with an undetectable viral load cannot pass on HIV. These attitudes did not significantly change between 2011 and 2013; a one-way MANOVA found no significant main effect for survey year (Wilks’ λ = 0.96, *F*[5, 209] = 1.59, *p* = 0.16, η_p_^2^ = 0.04). Control variables were not included in the analysis because there were no significant sociodemographic or behavioural differences between HIV-positive respondents in 2011 and 2013. The analysis was moderately powered to detect the effect (at 0.55).

### HIV treatment prevents transmission scale

Of the 1316 men who participated in the 2013 survey, 34 (2.6%) were classified as agreeing that HIV treatment prevents transmission (they scored ≥4 on the scale). The mean score for this scale was 2.11 (standard deviation [SD] = 0.79, median [Mdn] = 2.00, interquartile range [IQR] = 1.00). Table 3 shows the characteristics of these men compared with the rest of the sample. Multivariate logistic regression indicated that the belief that HIV treatment prevents transmission was independently associated with being HIV-positive (rather than HIV-negative), having an HIV-positive regular partner, and ever having received HIV post-exposure prophylaxis. Compared with men who had had no male sex partners in the past six months, men who had only one partner were less likely to believe that HIV treatment prevents transmission (but there was no difference between men with no partners and men with more than one recent partner).

**Table 3:** Factors associated with the belief that HIV treatment reduces transmission (scored 1–5).

		No (<4),n = 1282, %	Yes (≥4),n = 34, %	OR(95% CI)	*p*	Adjusted OR(95% CI)	*p*
Age	Mean in years (SD)	32.0 (11.1)	34.2 (12.0)	1.02 (0.98, 1.05)	0.32		
Sexual identity	Gay	91.7	85.3	Reference			
	Bisexual	6.4	8.8	1.48 (0.44, 4.97)	0.52		
	Other	1.9	5.9	3.38 (0.76, 14.98)	0.11		
HIV status	HIV-negative	73.8	58.8	Reference		Reference	
	Untested/unknown status	19.7	14.7	0.94 (0.35, 2.53)	0.90	1.20 (0.40, 3.60)	0.74
	HIV-positive	6.6	26.5	5.07 (2.24, 11.48)	<0.001	3.32 (1.34, 8.19)	0.009
Country of birth	Australia	77.1	76.5	Reference			
	Overseas	22.9	23.5	1.04 (0.47, 2.32)	0.93		
Residential location	Capital city	75.9	70.6	Reference			
	Other city	10.1	11.8	1.26 (0.43, 3.68)	0.68		
	Regional or rural area	14.0	17.6	1.35 (0.54, 3.35)	0.52		
Employment	Full-time	55.9	52.9	Reference			
	Part-time	10.1	11.8	1.23 (0.41, 3.68)	0.72		
	Student	24.3	23.5	1.02 (0.44, 2.38)	0.95		
	Unemployed/retired/other	9.7	11.8	1.28 (0.43, 3.86)	0.66		
Education	Up to Year 12 (high school)	28.6	20.6	Reference			
	Trade certificate	16.8	11.8	0.98 (0.28, 3.37)	0.97		
	Undergraduate degree	32.5	35.3	1.51 (0.59, 3.87)	0.39		
	Postgraduate degree	22.1	32.4	2.04 (0.78, 5.32)	0.15		
No. of male sex partners in past six months	None	10.1	17.6	Reference		Reference	
	One	25.0	5.9	0.13 (0.03, 0.67)	0.01	0.14 (0.03, 0.74)	0.02
	2–10	47.0	38.2	0.46 (0.17, 1.24)	0.13	0.22 (0.04, 1.22)	0.09
	>10	17.9	38.2	1.22 (0.45, 3.29)	0.69	0.41 (0.07, 2.49)	0.33
HIV status of regular male partner	No regular partner	44.2	44.1	Reference		Reference	
	HIV-negative	44.3	26.5	0.60 (0.26, 1.38)	0.23	1.00 (0.42, 2.42)	0.99
	Untested/unknown status	7.3	11.8	1.63 (0.53, 5.01)	0.40	2.02 (0.61, 6.64)	0.25
	HIV-positive	4.2	17.6	4.20 (1.57, 11.27)	0.004	3.02 (1.01, 9.08)	0.05
Anal intercourse with regular male partners (past six months)	No partner/no intercourse	34.9	41.2	Reference			
	Consistent condom use	18.2	2.9	0.14 (0.02, 1.05)	0.06		
	Any anal intercoursewithout condoms	46.9	55.9	1.01 (0.50, 2.04)	0.97		
Anal intercourse with casual male partners (past six months)	No partner/no anal intercourse	42.8	26.5	Reference		Reference	
	Consistent condom use	31.5	14.7	0.75 (0.25, 2.27)	0.62	1.03 (0.20, 5.45)	0.97
	Any anal intercoursewithout condoms	25.7	58.8	3.71 (1.67, 8.24)	0.001	3.31 (0.68, 16.11)	0.14
Ever received HIV post-exposure prophylaxis (PEP)	No	86.6	70.6	Reference		Reference	
	Yes	13.4	29.4	2.69 (1.26, 5.72)	0.01	2.49 (1.06, 5.88)	0.04

SD = standard deviation, OR = odds ratio, *p* = probability.

### Early HIV treatment is necessary scale

Of the 1316 participants in the 2013 survey, 945 (71.8%) were classified as agreeing that early treatment was necessary (they scored ≥4 on the scale). The mean score for this scale was 4.19 (SD = 0.77, Mdn = 4.33, IQR = 1.33). Table 4 shows the characteristics of these men compared with the rest of the sample. Multivariate logistic regression indicated that agreeing that early HIV treatment was necessary was independently associated with younger age. Participants who were HIV-positive and participants with an HIV-positive partner were much less likely to agree with the need for early treatment.

**Table 4:** Factors associated with the belief that early HIV treatment is necessary (scored 1–5).

		No (<4) n = 371 %	Yes (≥4) n = 945 %	OR (95% CI)	*p-*value	Adjusted OR (95% CI)	*p-*value
Age	Mean in years (SD)	35.0 (10.8)	30.9 (11.0)	0.97 (0.96, 0.98)	<.001	0.98 (0.97, 0.99)	.007
Sexual identity	Gay	91.1	91.7	Reference			
	Bisexual	6.5	6.5	0.99 (0.61, 1.62)	.97		
	Other	2.4	1.8	0.74 (0.33, 1.67)	.46		
HIV status	HIV-negative	71.4	74.2	Reference		Reference	
	Untested/unknown status	13.7	21.8	1.53 (1.09, 2.14)	.01	1.29 (0.86, 1.95)	.22
	HIV-positive	14.8	4.0	0.26 (0.17, 0.40)	<.001	0.35 (0.20, 0.59)	<.001
Country of birth	Australia	74.7	78.1	Reference			
	Overseas	25.3	21.9	0.83 (0.62, 1.09)	.18		
Residential location	Capital city	78.2	74.8	Reference			
	Other city	10.5	9.9	0.99 (0.66, 1.47)	.96		
	Regional or rural area	11.3	15.2	1.41 (0.97, 2.04)	.07		
Employment	Full-time	62.5	53.2	Reference		Reference	
	Part-time	9.4	10.5	1.30 (0.86, 1.98)	.21	1.19 (0.74, 1.91)	.47
	Student	18.1	26.7	1.73 (1.27, 2.37)	<.001	1.10 (0.73, 1.66)	.64
	Unemployed/retired/other	10.0	9.6	1.13 (0.75, 1.71)	.55	1.19 (0.73, 1.92)	.48
Education	Up to Year 12 (high school)	24.0	30.2	Reference		Reference	
	Trade certificate	18.3	16.0	0.69 (0.48, 1.01)	.05	0.95 (0.61, 1.47)	.82
	Undergraduate degree	32.9	32.5	0.79 (0.57, 1.08)	.14	0.85 (0.58, 1.23)	.38
	Postgraduate degree	24.8	21.4	0.69 (0.49, 0.97)	.03	0.97 (0.63, 1.47)	.87
No. of male sex partners in past six months	None	9.4	10.6	Reference		Reference	
	One	23.5	25.0	0.95 (0.60, 1.50)	.82	0.93 (0.54, 1.62)	.80
	2–10	42.0	48.7	1.03 (0.67, 1.58)	.88	1.00 (0.55, 1.80)	.99
	>10	25.1	15.8	0.56 (0.35, 0.89)	.01	0.67 (0.34, 1.29)	.23
HIV status of regular male partner	No regular partner	40.2	45.8	Reference		Reference	
	HIV-negative	43.9	43.8	0.87 (0.67, 1.13)	.31	0.86 (0.62, 1.19)	.36
	Untested/unknown status	7.5	7.3	0.85 (0.53, 1.37)	.50	0.64 (0.37, 1.11)	.12
	HIV-positive	8.4	3.1	0.32 (0.19, 0.55)	<.001	0.47 (0.25, 0.88)	.02
Anal intercourse with regular male partners (past six months)	No partner/no intercourse	33.7	35.7	Reference			
	Consistent condom use	15.4	18.7	1.15 (0.80, 1.65)	.44		
	Any anal intercourse without condoms	50.9	45.6	0.85 (0.65, 1.10)	.22		
Anal intercourse with casual male partners (past six months)	No partner/no anal intercourse	40.2	43.3	Reference		Reference	
	Consistent condom use	28.6	32.1	1.04 (0.78, 1.39)	.78	1.13 (0.73, 1.76)	.57
	Any anal intercourse without condoms	31.3	24.7	0.73 (0.55, 0.98)	.04	0.99 (0.62, 1.57)	.97
Ever received HIV post-exposure prophylaxis (PEP)	No	84.6	86.8	Reference			
	Yes	15.4	13.2	0.84 (0.60, 1.18)	.31		

SD = standard deviation, OR = odds ratio, *p* = probability.

## Discussion

Our repeated surveys of gay and bisexual men in Australia indicate relatively few changes in attitudes to HIV treatments and TasP over the last few years. Gay and bisexual men remain largely sceptical about the idea that HIV treatment prevents transmission, despite the release of the HPTN 052 results in between the surveys, although there has been a slight softening of attitudes among HIV-negative men [10]. HIV-negative and untested men remain uncertain about whether it is easy to take HIV treatments or not, while HIV-positive men tend to agree that being on treatment is straightforward.

Although very few men in our surveys (3%) clearly agreed that HIV treatment prevents transmission, HIV-positive men were three times more likely to believe this than HIV-negative men, as were men who had HIV-positive partners and men who had taken post-exposure prophylaxis. This echoes previous research on HIV treatment optimism, which found that HIV-positive men and men who engaged in practices that put them (or their partners) at increased risk of HIV transmission were more likely to believe in the preventative benefits of ART and an undetectable viral load [7], [16], [18], [19].

In contrast to the largely sceptical attitudes to the preventative benefits of ART, we found that the majority of gay and bisexual men (over 70%) supported the idea of early HIV treatment, specifically starting treatment immediately after diagnosis, not delaying treatment and going on treatment to protect one’s partners. Previous research has analysed prescribers’ attitudes to the early initiation of ART and HIV-positive people’s general attitudes to taking ART [10], [20], [21], but has rarely looked at community attitudes about when to start treatment. One recently published British study looked at attitudes to early ART among treatment naïve HIV-positive people, finding that over half would start treatment for health benefits or to protect partners [22]. In our study, support for early treatment was higher among younger men and noticeably lower among HIV-positive men and participants with HIV-positive partners. We suspect that this is because HIV-positive men (and those close to them) may be more aware of the risks and benefits of starting treatment than other men, and may be reticent about the idea of being encouraged to take treatment without consideration of these factors. This interpretation would be consistent with Australian ART prescribers’ views that decisions about when to commence HIV treatment should be primarily driven by a need to maximise individual wellbeing, and not solely for public health purposes [21].

Our results may appear contradictory to some – why are the majority of gay and bisexual men sceptical about TasP but supportive of early treatment? We think this indicates that while gay and bisexual men are well aware of the health benefits of ART for HIV-positive people [10], [20], they currently view TasP as an inadequate prevention strategy, or one that is unproven or partially efficacious. Other Australian research has found that most HIV-negative gay and bisexual men perceive any anal sex without condoms with HIV-positive men as risky, regardless of HIV viral load [23]. The concept of TasP suggests that it may be safe to have anal sex without condoms with HIV-positive men who have sustained viral suppression [3], but this contrasts with longstanding views of what counts as safe sex among Australian (and other) gay and bisexual men [24]. Therefore, while most of our participants would not rely on HIV treatment to prevent transmission (and are not very supportive of TasP), they appear to be comfortable supporting early treatment uptake. The support for early treatment appears to be based on ART’s perceived health benefits for the treated individual, while effects in preventing transmission are positioned as a subsidiary benefit for HIV-positive men and their partners.

Some constraints of our analysis should be borne in mind. Our surveys were cross-sectional and were not designed to identify causal relationships or to analyse changes in the attitudes of the same men over time. The profile of participants is similar to community samples of Australian gay and bisexual men in which HIV risk tends to be elevated, but is unlikely to be representative of all Australian gay and bisexual men [15], [25]. Our measures of the acceptability of TasP and the early initiation of treatment were derived from a range of attitudinal items about HIV treatments. We did not, however, give any background information to participants about the concept of TasP or current debates about when to initiate ART.

Our findings have a number of implications. HIV-negative and untested men in Australia remain uncertain about how easy it is to take HIV treatments, suggesting a need for community education about advances in antiretroviral therapy and contemporary experiences of taking ART. Gay and bisexual men remain largely sceptical about relying on ART to prevent HIV transmission. As we have noted before, this suggests that educating gay and bisexual men about advances in prevention science may be necessary to reassure HIV-positive people and their partners that sustained viral suppression appears to dramatically reduce the chance of transmission [10]. However, we previously noted that TasP does not appear to be a particularly acceptable strategy to gay and bisexual men, and community attitudes do not appear to have changed much in the last few years. This suggests a mismatch between strategic and policy efforts to maximise the preventative benefits of ART and community attitudes to acceptable forms of HIV prevention. It also suggests that changing community attitudes takes time. Highlighting the personal health benefits of HIV treatments for HIV-positive people rather than the public health benefits of preventing transmission appears to remain a more acceptable approach.

## Supporting Information

Dataset S1 **De-identified dataset.**
(SAV)
